# Intranasal Immunization with Nontypeable *Haemophilus influenzae* Outer Membrane Vesicles Induces Cross-Protective Immunity in Mice

**DOI:** 10.1371/journal.pone.0042664

**Published:** 2012-08-03

**Authors:** Sandro Roier, Deborah R. Leitner, Jeremy Iwashkiw, Kristina Schild-Prüfert, Mario F. Feldman, Georg Krohne, Joachim Reidl, Stefan Schild

**Affiliations:** 1 Institute of Molecular Biosciences, University of Graz, Graz, Austria; 2 Alberta Glycomics Centre, Department of Biological Sciences, University of Alberta, Edmonton, Alberta, Canada; 3 Division of Electron Microscopy, Biocenter of the University of Würzburg, Würzburg, Germany; Tulane University School of Medicine, United States of America

## Abstract

*Haemophilus influenzae* is a Gram-negative human-restricted bacterium that can act as a commensal and a pathogen of the respiratory tract. Especially nontypeable *H. influenzae* (NTHi) is a major threat to public health and is responsible for several infectious diseases in humans, such as pneumonia, sinusitis, and otitis media. Additionally, NTHi strains are highly associated with exacerbations in patients suffering from chronic obstructive pulmonary disease. Currently, there is no licensed vaccine against NTHi commercially available. Thus, this study investigated the utilization of outer membrane vesicles (OMVs) as a potential vaccine candidate against NTHi infections. We analyzed the immunogenic and protective properties of OMVs derived from various NTHi strains by means of nasopharyngeal immunization and colonization studies with BALB/c mice. The results presented herein demonstrate that an intranasal immunization with NTHi OMVs results in a robust and complex humoral and mucosal immune response. Immunoprecipitation revealed the most important immunogenic proteins, such as the heme utilization protein, protective surface antigen D15, heme binding protein A, and the outer membrane proteins P1, P2, P5 and P6. The induced immune response conferred not only protection against colonization with a homologous NTHi strain, which served as an OMV donor for the immunization mixtures, but also against a heterologous NTHi strain, whose OMVs were not part of the immunization mixtures. These findings indicate that OMVs derived from NTHi strains have a high potential to act as a vaccine against NTHi infections.

## Introduction

*Haemophilus influenzae* is a Gram-negative coccobacillus that commonly colonizes the human respiratory tract as a commensal or pathogen. This bacterium can be differentiated into typeable and nontypeable strains based on the presence or absence of a polysaccharide capsule. Encapsulated strains are divided into six capsular serotypes (a–f), with serotype b (Hib) being the most common associated with human disease. Infections caused by Hib strains range mainly from meningitis and acute epiglottitis to sepsis. In contrast, nonencapsulated and therefore nontypeable *H. influenzae* (NTHi) strains generally cause pneumonia, sinusitis, and otitis media [1], [2], [3]. Additionally, NTHi strains are one of the most common bacterial cause of exacerbations in patients suffering from chronic obstructive pulmonary disease (COPD) [4], [5]. According to the latest WHO estimates, over 3 million people died of COPD in 2004 and it is predicted that COPD will become the third leading cause of death worldwide by 2030 [6].

Since the introduction of capsular polysaccharide conjugate vaccines against Hib in the late 1980s, invasive Hib diseases have been dramatically reduced in many developed countries [7]. In contrast, invasive diseases caused by NTHi infections have been steadily recognized since Hib vaccination began and have become the most frequent cause of an invasive *H. influenzae* disease in some regions [8], [9], [10], [11]. Due to this altered epidemiology of invasive *H. influenzae* infections, particularly acute otitis media, and the increasing burden of COPD-related morbidity and mortality, there is a high demand for an effective NTHi vaccine. Besides the fact that NTHi strains have no conserved capsule, the key limitations for vaccine development are the high genetic heterogeneity of NTHi strains as well as the enormous antigenic variability of several surface-exposed antigens [1], [12], [13]. Therefore, vaccine development has focused on highly conserved structures of outer membrane proteins (OMPs), lipooligosaccharide (LOS), or pili. Among the most promising vaccine candidates are OMPs like P2, P4, P5, P6, protein D and E, since these antigens are highly immunogenic and represent abundant surface proteins in many NTHi isolates [13], [14], [15], [16], [17], [18]. In addition, also LOS conjugate vaccines against NTHi have been investigated [19], [20]. In order to be immunogenic the detoxified LOS has to be conjugated to a carrier, such as the tetanus toxoid, high-molecular-weight proteins or P6 [20], [21]. Recent studies indicate that even P6, which was believed to be one of the most conserved OMPs in NTHi, is not conserved in all NTHi strains and may not be surface exposed [22], [23]. Thus, focusing on single antigens might not be the best approach for an effective NTHi vaccine. Instead, presenting a combination of multiple heterologous antigens to the immune system could increase the efficacy of a vaccine against heterologous NTHi strains. In this regard, outer membrane vesicles (OMVs) could be considered as a new promising vaccine candidate.

OMVs are natural secretion products of Gram-negative bacteria. They are released when parts of the outer membrane (OM) bulge and pinch off in the form of spherical and bilayered vesicles. These vesicles range in size from 10 to 300 nm in diameter and consist mainly of OM components, such as phospholipids, OMPs, and lipopolysaccharide (LPS) or LOS. Additionally, OMVs contain periplasmic components, which are trapped in the lumen of OMVs at the vesiculation process [24], [25], [26], [27]. Due to the release from the OM, OMVs reflect the natural composition of the OM [24], [28], [29], [30]. Therefore, OMVs carry multiple native bacterial antigens, which, combined with their multi-immunogenic and self-adjuvant properties, make them of particular interest for vaccine development [31]. An additional important feature is that the surface-exposed membrane antigens of OMVs maintain their physico-chemical stability [32], [33]. For these reasons, the immunogenic and protective properties of OMVs have been tested and proven for several bacterial species, e. g. *Vibrio cholerae*, *Salmonella typhimurium*, *Borrelia burgdorferi*, *Bordetella pertussis*, and *Porphyromonas gingivalis* [32], [34], [35], [36], [37], [38], [39]. However, the greatest success has been achieved with OMVs derived from *Neisseria meningitidis*. So far, OMV vaccines are the only protective formulation against *N. meningitidis* serogroup B and have been extensively used in several countries demonstrating both safety and efficacy [33], [40].

To date, all Gram-negative bacteria that have been investigated for secretion of vesicles are able to naturally release OMVs [25], [27]. The release of OMVs by *H. influenzae* was first described for a nonencapsulated serotype d strain correlating with the loss of competence [41], [42]. The presence of OMVs in NTHi cultures has been documented by two studies using electron microscopy [16], [43]. A recent study identified 142 proteins present in OMVs derived from NTHi strain 86-028NP [44]. Consistent with OMV proteomes of other Gram-negative bacteria and the current models of OMV biogenesis mainly periplasmic proteins, OMPs or OM associated proteins have been elucidated.

In the present study the release of OMVs from several NTHi strains was analyzed by electron microscopy, protein profile comparisons and identification of abundant cargo proteins. Furthermore, we examined the capacity of OMVs derived from NTHi strains to act as a potential vaccine against NTHi infections by means of nasopharyngeal immunization and colonization studies with BALB/c mice. Therefore, we investigated the humoral, mucosal, and protective immune responses against homologous and heterologous NTHi strains after intranasal immunization with NTHi OMVs.

## Materials and Methods

### Ethics statement

Female BALB/c mice (Charles River Laboratories) were used for all immunization experiments in strict accordance with the recommendations in the Guide for the Care and Use of Laboratory Animals of the National Institutes of Health, the national “Bundesgesetzblatt fuer die Republik Oesterreich”. The corresponding animal protocol (39/158 ex 2000/10), has been approved by the Austrian Federal Ministry of Science and Research Ref. II/10b and the Committee on the Ethics of Animal Experiments of the University of Graz. Mice were housed with food and water *ad libitum* and monitored under the care of full-time staff and in accordance with the rules of the Institute of Molecular Biosciences at the University of Graz. All animals were acclimated for 1 week before any procedures were carried out and were 9 weeks old at the start of the immunization.

### Bacterial strains and growth conditions

The NTHi strains 1479, 2019, 3198, 5657, 7502, and 9274 were kindly provided by Michael A. Apicella, University of Iowa, USA. Additionally, the spontaneous streptomycin-resistant (Sm^r^) derivative AC53 of *V. cholerae* strain E7946 was used [45]. All NTHi strains are clinical isolates from the sputum or middle ear fluid of human patients and have been described previously [21], [46]. Spontaneous Sm^r^ derivatives 1479-R, 2019-R, 3198-R, 7502-R, and 9274-R of the respective NTHi strains as well as 5657 were used in all experiments. Sm^r^ derivatives allowed the positive selection throughout the study including the challenge experiment and were generated by plating overnight cultures of the respective strains on brain heart infusion (BHI) agar supplemented with streptomycin. After 48 h, Sm^r^ colonies were recovered, purified, and compared with their respective donor wild-type strains for their OM and OMV protein profiles as well as for their growth kinetics. In all cases, no obvious differences were observed (data not shown).

Bacteria were grown at 37°C with aeration in LB broth or on LB agar in the case of *V. cholerae* as well as in BHI broth or on BHI agar supplemented with NAD and hemin-solution (stock-solution containing a mixture of hemin, L-histidine, and triethanolamine) in the case of NTHi. When appropriate, streptomycin was added. Supplements were used in the following final concentrations: NAD, 10 µg/ml; hemin, 20 µg/ml; L-histidine, 20 µg/ml; triethanolamine, 0,08%; and streptomycin, 100 µg/ml.

### Multilocus sequence typing (MLST)

MLST analysis of the NTHi isolates was performed as described in the MLST database (http://haemophilus.mlst.net) [47]. The sequences of seven genes for each isolate were submitted to the international MLST database to obtain the allelic profile of each sample as well as the sequence type (ST) of each isolate. In case of the *mdh* locus of NTHi strain 1479, a new allelic number and a new MLST sequence type were assigned by a curator of the international database. The MLST results have been added to the database and are available online (http://haemophilus.mlst.net/).

### Preparation of OMPs and whole-cell lysates (WCL)

Proteins of the OM were prepared according to Carlone et al. [48]. Briefly, cells from 10 ml of the respective NTHi overnight culture were harvested by centrifugation (3,200× g, 10 min, 4°C). The cell pellet was washed once in HEPES buffer (10 mM, pH 7.4) and resuspended in 1 ml HEPES buffer (10 mM, pH 7.4) with protease inhibitor (Roche, Complete EDTA-free protease inhibitor cocktail, 1 tablet per 50 ml). Cells were disrupted by sonification (six bursts, 10 sec each) using a Branson Ultrasonics sonifier S-250A. Unbroken cells were removed by centrifugation (15,600× g, 2 min, 4°C). The supernatant containing the OMPs was transferred into a new tube and centrifuged again (15,600× g, 30 min, 4°C). The membrane pellet was resuspended in 0.4 ml HEPES buffer (10 mM, pH 7.4). To solubilize the cytoplasmic membrane, 0.4 ml HEPES buffer (10 mM, pH 7.4) with 2% sarcosyl was added and incubated at room temperature (RT) for 30 min with intermittent mixing. After centrifugation (15,600× g, 30 min, 4°C), the pellet containing the OMPs was washed once with 0.5 ml HEPES buffer (10 mM, pH 7.4) and finally resuspended in 50 µl HEPES buffer (10 mM, pH 7.4). Purified OMPs were stored at −20°C.

WCL were prepared by sonification similar to the preparation of OMPs as described above. After removal of unbroken cells by centrifugation, the remaining supernatant served as WCL. Purified WCL were stored at −20°C.

The protein concentrations of OMP and WCL preparations were determined by photometric measurements of the absorbances at 260 nm and 280 nm using a Beckman Coulter DU730 spectrophotometer in combination with a TrayCell (Hellma) and the Warburg-Christian equation.

### Preparation of OMVs

*V. cholerae* OMVs were isolated as previously published [34]. NTHi OMVs were isolated accordingly, except for the following modifications: 500 ml BHI broth was inoculated with 5 ml of the respective NTHi overnight culture and grown to late exponential phase for 13 h. Bacterial cells were pelleted by two subsequent centrifugation steps (6,400× g, 10 min, 4°C and 16,000× g, 6 min, 4°C). The supernatant containing the OMVs was consecutively filtered through 0.45 µm and 0.2 µm pore size filters to ensure complete removal of remaining bacteria. 0.2 ml of the filtrate was plated on a BHI agar plate and incubated 48 h to confirm the absence of viable bacteria. In all preparations, no colonies were observed. The filtrate was stored at 4°C and within the next two days OMVs were pelleted from the filtrate by ultracentrifugation (144,000× g, 4 h, 4°C) using a Beckman Coulter Optima L-100 XP ultracentrifuge and a SW 32 Ti rotor. The OMV pellet was resuspended in approximately 150 µl phosphate-buffered saline (PBS, pH 7.4) and stored at −70°C. The protein concentrations of the OMV preparations were determined as described above and adjusted to 2.5 µg/µl using PBS (pH 7.4).

### Immunization protocol and experimental setup

**Figure 1:**
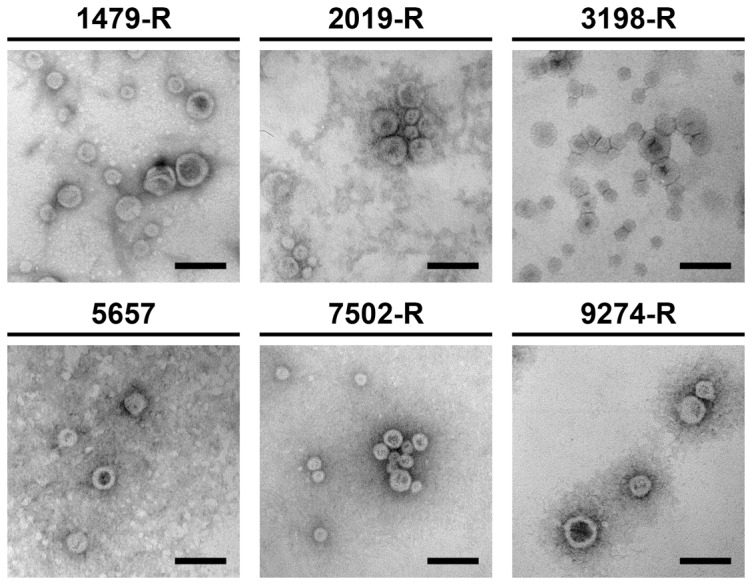
OMV and OM protein profiles of heterologous NTHi strains. Protein profile comparisons of OMV and OM preparations from the heterologous NTHi strains (A) 9274-R, 2019-R, and 1479-R, as well as (B) 7502-R, 5657, and 3198-R are shown. Samples (approx. 4 µg protein each) were separated by SDS-PAGE (12,6% gels) and protein bands were visualized according to Kang et al. [59]. Lines to the left indicate the molecular masses of the protein standards in kDa.

For the immunization with OMVs derived from various NTHi strains or *V. cholerae*, mice were divided into different immunization groups. These groups received either immunization mixture 1 (IM-1) consisting of OMVs derived solely from NTHi 2019-R (Table 1) via the intranasal or intraperitoneal (i.p. IM-1) administration route, immunization mixture 2 (IM-2) consisting of equally mixed OMVs derived from NTHi 2019-R, 9274-R, and 1479-R (Table 1) via the intranasal administration route, or OMVs derived from *V. cholerae* AC53 via the intranasal administration route (i.n. Vch-OMV). NTHi strain 2019-R was chosen as the sole donor for OMVs used in IM-1, because it was extensively used in several *in vivo* studies, it is the prototype strain for LOS group II, and its LOS structure has been identified [19], [49], [50], [51], [52], [53], [54]. In addition to 2019-R OMVs, also OMVs derived from NTHi strains 9274-R and 1479-R were used in IM-2 to increase the antigen complexity of the immunization mixture. These strains were chosen, because they have been classified into different LOS groups (type I, II and III) and demonstrate a high OM heterogeneity [19], [46], [49]. The LOS structure of NTHi strain 9274-R was identified [55] and differs significantly from NTHi strain 2019-R [52]. Besides the LOS structure, the diversity of these strains is also indicated by their heterogeneous OMV protein profiles and allocation into different ST (Fig. 1A and Table 2). Antibody titers in serum to OMVs were monitored at four time points before (day 0), during (day 14 and 28), and after (day 39) the immunization period by using an indirect ELISA and plates coated with OMVs derived from various NTHi strains serving as antigen. ELISA plates coated with 2019-R OMVs were used to determine the immunoglobulin responses against a homologous strain, whose surface antigens were presented (known) to the immune systems of mice immunized with either IM-1 or IM-2. In contrast, OMVs derived from the heterologous NTHi strains 3198-R, 5657, and 7502-R [19], [46], [49] were neither present in IM-1 nor IM-2. The strains 5657 and 7502-R are the prototype strains for the LOS groups IV and V, respectively. Thus, ELISA plates coated with OMVs derived from these strains were used to determine the immune responses against heterologous strains, whose surface antigens were not presented (unknown) to the immune systems of the immunized mice. In contrast, strain 3198-R exhibits OM heterogeneity in the protein profile, but belongs to the LOS group III like the strain 9274-R, which was used as one donor for OMVs provided in IM-2. Therefore, ELISA plates coated with OMVs derived from strain 3198-R reflect a heterologous strain, whose surface protein antigens were unknown, while a similar LOS structure has been presented to the immune systems of the mice immunized with IM-2. In the case of mice immunized with IM-1, surface protein and LOS antigens of 3198-R were unknown to the immune system.

**Table 1:** Experimental setup of the immunization mixtures and NTHi strains used in this study with respect to their relevance for the immune systems of the immunized mice.

		Relevance for the immune systems of immunized mice			
Immunization mixture (IM)	OMV donor strain for the IM	Known^a^ LOS (LOS group^b^)	Known surface proteins (OMP subtype^c^)	Unknown^a^ LOS (LOS group)	Unknown surface proteins (OMP subtype)
IM-1	2019-R d	2019-R (II)	2019-R (2)	1479-R (I)	1479-R (1)
				3198-R (III)	3198-R (8)
				9274-R (III)	9274-R (n.d.^e^)
				5657 (IV)	5657 (7)
				7502-R (V)	7502-R (3)
IM-2	1479-R	1479-R (I)	1479-R (1)	5657 (IV)	5657 (7)
	2019-R	2019-R (II)	2019-R (2)	7502-R (V)	7502-R (3)
	9274-R	9274-R (III)	9274-R (n.d.)		3198-R (8)
		3198-R (III)			

a Known and unknown refer to the presence or absence of the particular LOS or surface proteins in the immunization mixtures, respectively.

b LOS group classification as previously published [19], [49].

c According to the OMP subtype classification developed by Murphy et al. [46] and the heterologous protein profiles shown in Fig. 1.

d Strains underlined were used for challenge.

e Not determined by Murphy et al. [46].

**Table 2:** Comparative MLST analysis of the NTHi strains used in this study.

	Allelic profile^a^							
Strain	*adk*	*atpG*	*frdB*	*fucK*	*mdh*	*pgi*	*recA*	ST^a^
1479	68	43	55	11	216	58	18	1003
2019	28	14	1	1	62	13	1	321
3198	33	8	16	16	49	2	3	107
5657	1	8	1	14	22	14	13	145
7502	14	7	13	1	136	83	83	380
9274	1	1	1	14	59	14	5	243

a According to the MLST database (http://haemophilus.mlst.net).

For all immunization experiments a nonvaccinated control group of sham immunized mice with PBS (pH 7.4) were housed in parallel with vaccinated mice for the duration of the experiment. No adjuvant was used for the entire study. To avoid potential effects by coprophagia mice of each immunization groups as well as the control mice were kept in separated cages. Mice were intranasally (25 µg OMVs in 10 µl PBS, 5 µl per nostril) or intraperitoneally (2 µg OMVs in 100 µl PBS) immunized at days 0, 14, and 28. This immunization doses were based on previously published immunization studies using OMVs derived from *V. cholerae* and *N. meningitidis* [34], [35], [56], [57]. Mice were briefly anesthetized by inhalation of 2.5% isoflurane gas prior to all immunizations. None of the animals died throughout the immunization study and no significant differences in consumption of food and water was detected between cages harbouring vaccinated mice and control mice. Overall two independent rounds of immunization for each immunization group were performed with at least 3 mice per round and different batches of OMV preparations, respectively. Comparison of the data from the independent immunization rounds revealed no differences in the induced immune response or protection for the respective immunization groups.

### Collection and preparation of blood and stool samples

Blood samples were collected by lateral tail vein nick at day 0, 14, and 28, as well as by cardiac puncture at day 39. Additionally, three to five freshly voided fecal pellets per mouse were collected at day 39. Samples were processed as described previously [34], [35]. Serum samples and extracted Igs from the fecal pellets were stored at −70°C.

### Challenge with NTHi

All vaccinated and nonvaccinated control mice were challenged with NTHi at day 38 for 24 h. Mice were inoculated with approximately 5×10^5^ CFU of either NTHi 2019-R or NTHi 3198-R. To prepare the inoculum, the respective NTHi strains were grown in BHI broth to an optical density at 490 nm (OD_490_) of 1. Cells were harvested by centrifugation (2,300× g, 5 min, RT), resuspended in PBS (pH 7.4), and adjusted to an OD_490_ of 1.1 (equivalent to approximately 1×10^9^ CFU/ml). Subsequently, 1∶10 dilutions in PBS (pH 7.4) were prepared. The first 1∶10 dilution was mixed 1∶1 with PBS (pH 7.4) to obtain the inoculum (approximately 5×10^7^ CFU/ml). In parallel appropriate dilutions were plated on BHI plates supplemented with streptomycin and incubated at 37°C for two days to determine the CFU/ml of the inoculum by back-calculating to the original suspension. Prior to challenge, mice were briefly anesthetized by inhalation of 2.5% isoflurane gas. Then mice were intranasally inoculated with approximately 5×10^5^ CFU using 10 µl (5 µl per nostril) of the inoculum. After 24 h, the mice were sacrificed and the nasopharynx from each mouse was removed by dissection. The nasopharynx was mechanically homogenized in BHI broth with 15% glycerol and appropriate 1∶10 dilutions were plated on BHI plates supplemented with streptomycin. After incubation at 37°C for two days, the colonization rates in CFU/nasopharynx were determined by back-calculation to the original volume of the homogenized nasopharynges.

### Quantitation of antibodies

Immunoglobulin A (IgA), IgG1, and IgM antibodies as well as half-maximum total Ig titers (IgA, IgG, and IgM) to OMVs were quantified by indirect enzyme-linked immunosorbent assay (ELISA) using 96-well ELISA microplates (BD Falcon) essentially as described previously [34], [35]. ELISAs were performed by using appropriate OMVs (5 µg/ml in PBS, pH 7.4) as coating antigens as well as appropriate Ig isotype standards (BD Biosciences) and horseradish peroxidase-conjugated goat anti-mouse secondary antibodies (Southern Biotech). For detection, the TMB peroxidase substrate reagent set (BioLegend) was used according to the manufacturer's instructions. Optical densities were read at 450 nm with a FLUOstar Omega microplate reader (BMG Labtech). Antibody titers were calculated using values from appropriate dilutions of the test samples and a log-log regression calculated from at least four dilutions of the isotype standards. Half-maximum total Ig titers were calculated by plotting the log of the reciprocal dilutions of mouse sera against the resulting absorbances to create sigmoidal curves, which were used to determine the reciprocals that gave half of the theoretical maximum optical density.

### SDS-PAGE and immunoblot analysis

In order to analyze the protein content of the OM, WCL, and OMVs, proteins were separated by sodium dodecyl sulfate-polyacrylamide gel electrophoresis (SDS-PAGE) [58] in combination with 12.6% gels using the Prestained Protein Marker Broad Range (New England Biolabs) as a molecular mass standard. Protein bands were visualized according to Kang et al. [59].

Immunoblot analysis was performed as described previously [34]. Chemiluminescence detection was performed by using the Immun-Star™ WesternC™ Kit (Bio-Rad Laboratories) and subsequent exposure in a ChemiDoc XRS system (Bio-Rad Laboratories) in combination with Quantity One software (Bio-Rad Laboratories).

### Immunoprecipitation

Immunoprecipitation was performed by using the Dynabeads® Protein G Immunoprecipitation Kit (Invitrogen) according to the manufacturer's manual. To avoid mouse-specific variations, sera collected on day 39 from all mice immunized with IM-1 were pooled and 16 µl of this mixture was used for binding of the antibodies to the beads. 16 µl of pooled serum collected on day 39 from the nonvaccinated control mice served as a negative control. 100 µl of an OMP preparation (2 µg/µl) from strain 2019-R1 was used as antigen. Proteins in the immunoprecipitations were separated by SDS-PAGE and analyzed by mass spectrometry.

### MALDI TOF-TOF MS and MS/MS analysis

Protein bands from the OMV and immunoprecipitation samples were excised and in-gel digested using sequencing grade modified trypsin (Promega) [60]. Peptide fragments were eluted from the gel piece, desalted using ZipTipC_18_ (Millipore) according to the supplier protocol, resuspended in 0.1% formic acid, spotted onto a Bruker Daltonics MTP AnchorChip™ 800/384 target and air dried. 0.42 µL of α-cyano-4-hydroxy cinnamic acid matrix solution (CHCA) was spotted on top and air dried. The matrix solution was prepared by diluting 36 µL of saturated matrix solution in 0.1% TFA in 90∶10 ACN∶H_2_O to 800 µL final volume using 0.1% TFA in 85∶15 ACN∶H_2_O, containing 1 mM ammonium phosphate. Mass spectra were obtained in the positive reflectron mode of ionization using a Bruker Daltonics (Bremen, Germany) UltrafleXtreme MALDI TOF/TOF mass spectrometer. The MS and MS/MS spectra were obtained in an automated mode of operation. For MS/MS analysis the CID (collision-induced dissociation) gas was turned off. The FlexAnalysis, WARP-LC, ProteinScape software packages provided by the manufacturer and an in house MASCOT server were used for analysis of the mass spectra.

### Transmission electron microscopy

For OMV visualization by electron microscopy, vesicles were purified as described above and adjusted to a final concentration of 2.5 µg/µl using PBS (pH 7.4). Samples were fixed in 0.5% glutaraldehyde (10 mM cacodylate pH 7.2, 10 mM KCl, 0.5 mM MgCl_2_) for 5 min and subsequently diluted 1∶100 in H_2_O. 12 µl of the dilutions were allowed to adsorb onto glow-discharged 300-mesh carbon coated copper grid for 1 min followed by negative staining with aqueous uranyl acetate (0.5%). The micrographs were recorded at an accelerating voltage of 80 kV using a EM10 electron microscope (Zeiss, Oberkochen, Germany). The photographic negatives were digitalized by scanning and processed using Adobe Photoshop (Adobe Systems Incorporated).

### Statistical analysis

Data were analyzed using the Mann-Whitney U test or a Kruskal-Wallis test followed by *post hoc* Dunn's multiple comparisons. Differences were considered significant at *P* values of <0.05. For all statistical analyses, GraphPad Prism version 4.0a for Mac OS X (GraphPad Software) was used.

## Results

### NTHi strains release OMVs

To investigate whether the NTHi strains used in this study are able to release OMVs, we adapted an established method to isolate *V. cholerae* OMVs derived from culture supernatants [34]. Six heterologous NTHi strains were tested for OMV production. These included the five prototype NTHi strains 1479-R (type I), 2019-R (type II), 3198-R (type III), 5657 (type IV) and 7502-R (type V) based on the LOS typing system developed by Campagnari et al. [49]. Additionally, we used the NTHi strain 9274-R (type III), which has been used for various LOS vaccination studies [19], [20], [21], [61], [62], [63]. Phylogenetic relationship of the NTHI strains was characterized by MLST analysis to prove the diversity of the NTHi strains. In the case of NTHI strains 2019 and 9274, the MLST data was already available in the respective database (http://haemophilus.mlst.net/). We identified the allelic profile and sequence types (ST) for the NTHi strains 1479, 3198, 5657 and 7502, which subsequently allowed a comparison of all six NTHi strains (Table 2). The isolates exhibited high variation in their allelic profiles. Consequently all strains are allocated into different ST. The best match was obtained for NTHi strains 5657 and 9274 with only 3 identical alleles out of 7, while all other comparisons revealed less then 3 identical alleles. Thus, the six strains used in this study are highly heterologous.

**Figure 2:**
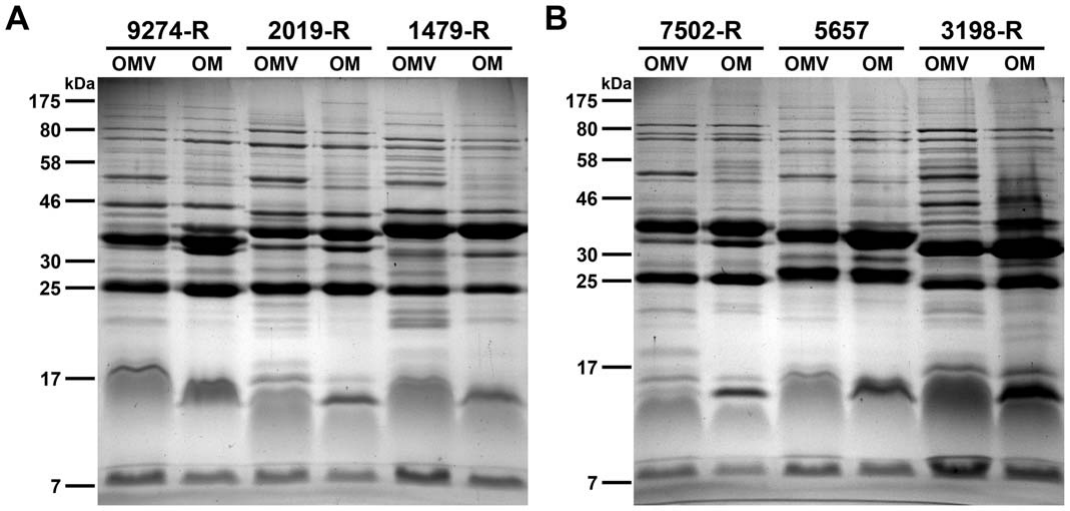
OMV visualization by electron microscopy. Transmission electron microscopy of purified OMVs derived from NTHi strain 1479-R, 2019-R, 3198-R, 5657, 7502-R and 9274-R negatively stained with uranyl acetate. The scale bars represent 100 nm.

Cell-free vesicle samples of these heterologous NTHi strains were isolated from late log-phase cultures using filtration and ultracentrifugation steps (see Materials and Methods for details). Purified vesicles were analyzed by transmission electron microscopy (Fig. 2). All samples contained multiple spherical vesicles, the majority of which ranged between 20 and 80 nm in size. The protein patterns of the purified OMVs were analyzed and compared with the respective OM preparations by SDS-PAGE in combination with Kang's staining method (Fig. 1). All strains tested revealed similar protein profiles of the respective OMV and OM samples, indicating that the abundant proteins of the OM are also present in the derived OMVs. Some protein bands are over- or underrepresented in the OMV preparations compared to the OM preparations, but such enrichments and exclusions of proteins in OMVs have been described before for other bacteria [64], [65], [66], [67], [68]. The surface protein heterogeneity of these NTHi strains is highlighted by their heterogeneous OMV or OMP profiles, especially by the variable migration patterns of the two most abundant protein bands located between 25 and 46 kDa. Thus, all strains used in this study reflect the typical variation in the OMP patterns, which is a hallmark of NTHi strains [46], [69], [70], [71], [72], [73], [74].

**Figure 3:**
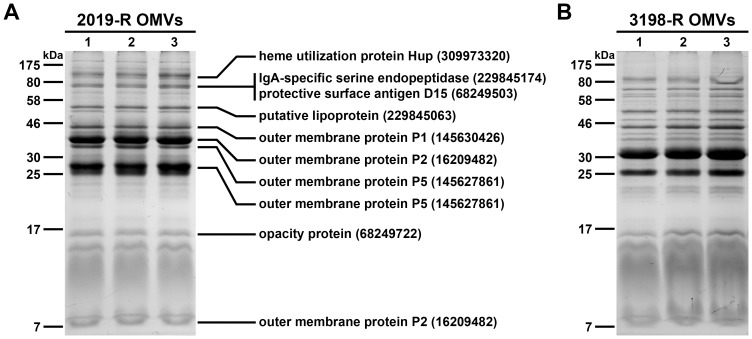
OMV protein profiles of independent preparations. Shown are protein profile comparisons of three independent OMV preparations from the NTHi strains 2019-R (A) and 3198-R (B). Samples (approx. 4 µg protein each) were separated by SDS-PAGE (12,6% gels) and protein bands were visualized according to Kang et al. [59]. Lines to the left indicate the molecular masses of the protein standards in kDa. Proteins identified by mass spectrometry from NTHi 2019-R OMVs are indicated with their respective position on the gel, protein identities and accession numbers on the right.

To exclude variability within OMV preparations of a respective NTHi strain, we compared the protein profiles from OMV samples isolated from independent cultures of each NTHi strain. Representative examples are provided in Fig. 3 showing three independent preparations of OMVs derived from NTHi strain 2019-R (Fig. 3A) and 3198-R (Fig. 3B), which are the most important samples used in this study. For both strains, similar patterns and intensities of the protein bands in these OMV preparations derived from three independent cultures can be observed. Such low variations in independent OMV preparations were also observed for all other strains used in this study (data not shown). This indicates that at least the protein composition of the OMVs is highly reproducibility and a constant quality can be achieved in independent OMV preparations.

In order to characterize the protein cargo of the vesicles, the most intensive protein bands of the OMVs derived from NTHi strain 2019-R were excised and subjected to tryptic digestion and mass spectrometry. The analysis identified eight proteins of the NTHi 2019-R OMVs, with the OMPs P2 and P5 including their smaller sized degradation products being the most abundant ones (Fig. 3A). Additionally, OMPs or OM associated proteins like the heme utilization protein, the IgA-specific serine endoprotease, the protective surface antigen D15, a putative lipoprotein, the OMP P1 and the opacity protein have been identified. Consistent with our data, these proteins were also found in the proteome of OMVs derived from NTHi strain 86-028NP [44].

In summary, our data confirms that NTHi strains in fact release OMVs during growth and that these OMVs can be reproducibly isolated by a simple protocol based on filtration and ultracentrifugation.

### Immunization with NTHi OMVs induces humoral and mucosal immune responses

Mice were intranasally immunized with NTHi OMVs at day 0, 14, and 28 using the two immunization mixtures IM-1 and IM-2, respectively. Table 1 provides a schematic overview of the experimental setup and a detailed description of the design as well as the composition of the immunization mixtures can be found in the Materials and Methods (“Immunization protocol and experimental setup”). Briefly, IM-1 contained only OMVs derived from NTHi strain 2019-R1, whereas IM-2 was composed of equally mixed OMVs derived from NTHi strain 2019-R 9274-R and 1479-R to increase the antigen complexity of the immunization mixture. Antibody titers in serum to OMVs were monitored by ELISA using OMVs derived from various NTHi strains as coating antigen. ELISA plates coated with 2019-R OMVs were used to determine the immunoglobulin responses against a homologous strain, whose surface antigens were presented (known) to the immune systems of mice immunized with either IM-1 or IM-2. In contrast, OMVs derived from the heterologous NTHi strains 3198-R, 5657, and 7502-R were neither present in IM-1 nor IM-2. Thus, ELISA plates coated with OMVs derived from these strains were used to determine the immune responses against heterologous strains, whose surface antigens were not presented (unknown) to the immune systems of the immunized mice.

**Figure 4:**
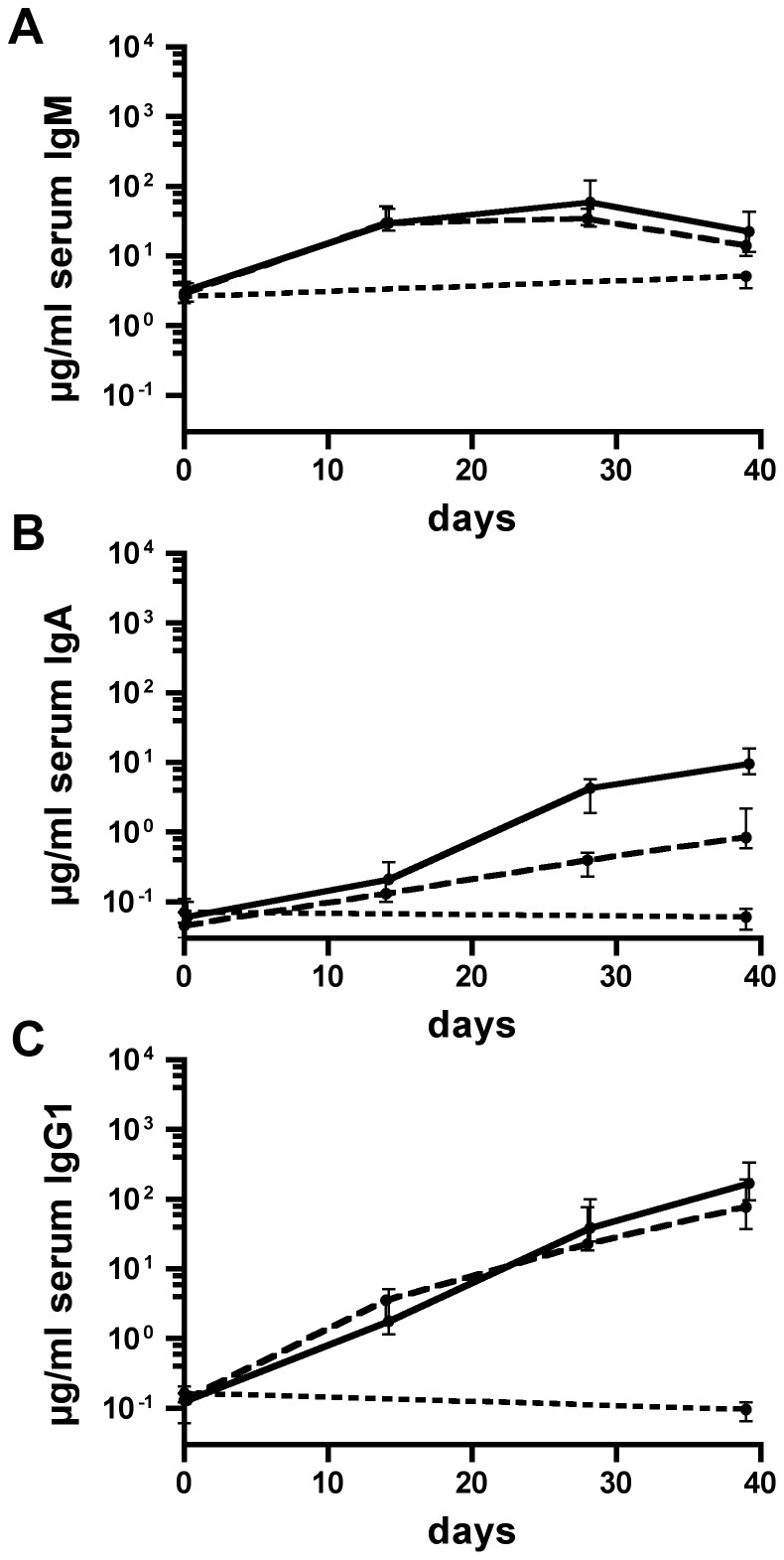
Temporal immune responses to OMVs derived from NTHi strain 2019-R. Shown are the median titers over time of IgM (A), IgA (B), and IgG1 (C) antibodies to 2019-R OMVs in sera from mice intranasally immunized with either IM-1 (solid line) or IM-2 (dashed line) as well as in sera from nonvaccinated control mice (dotted line) (n = 20 for each group). The error bars indicate the interquartile range of each data set for each time point.

**Figure 5:**
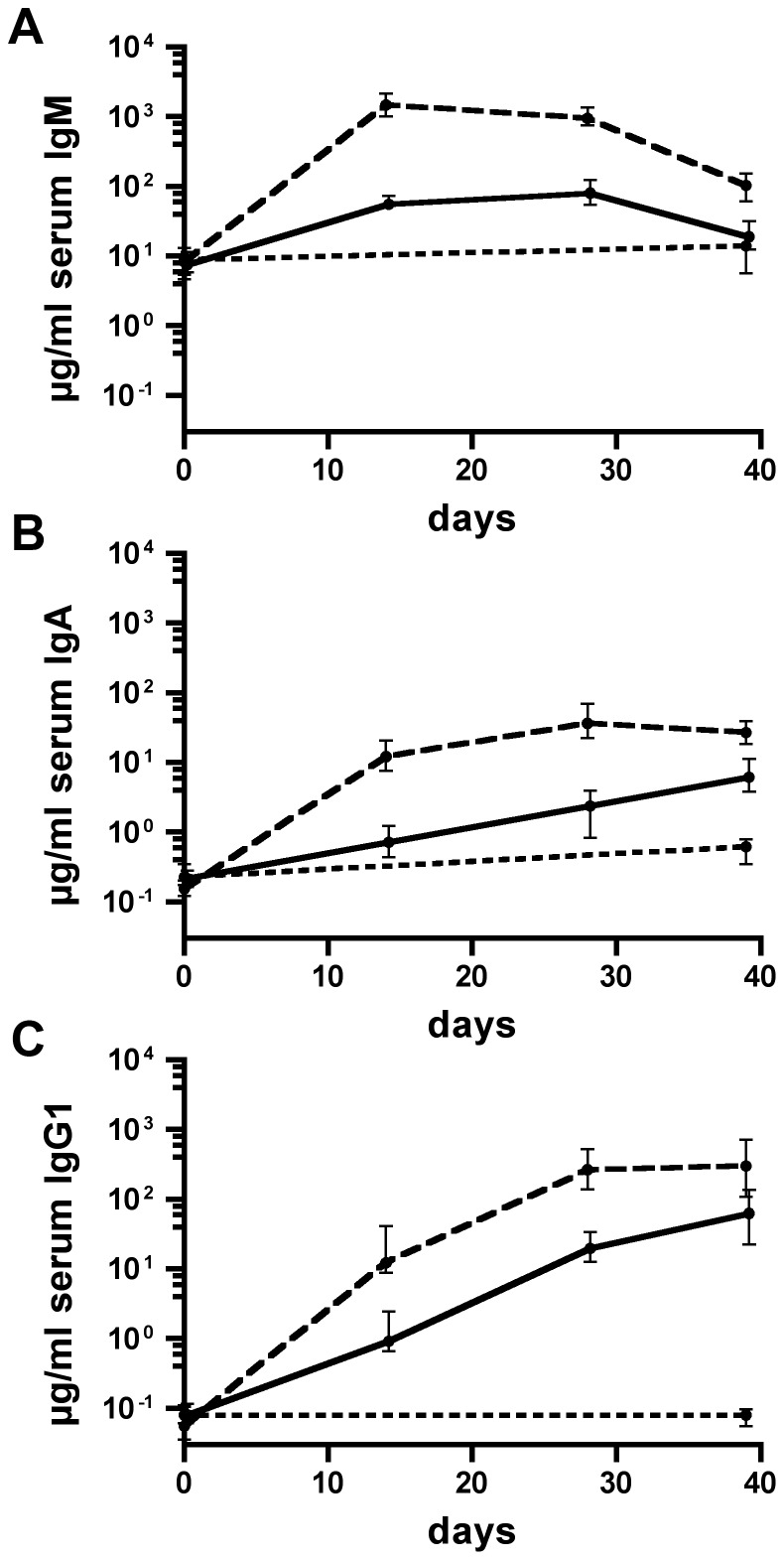
Temporal immune responses to OMVs derived from NTHi strain 3198-R. Shown are the median titers over time of IgM (A), IgA (B), and IgG1 (C) antibodies to 3198-R OMVs in sera from mice intranasally immunized with either IM-1 (solid line) or IM-2 (dashed line) as well as in sera from nonvaccinated control mice (dotted line) (n = 20 for each group). The error bars indicate the interquartile range of each data set for each time point.

The temporal IgM, IgA, and IgG1 responses to OMVs derived from 2019-R or 3198-R are shown in Fig. 4 and Fig. 5, respectively. The IgM, IgA, and IgG1 titers of the nonvaccinated control group were determined only for day 0 and 39, since in general no significant increases were observed within this period (*P*>0.05; Mann-Whitney U test). At day 0, the median isotype-specific antibody titers to OMVs derived from 2019-R or 3198-R were relatively low and showed no significant differences between the three groups (*P*>0.05; Kruskal-Wallis test and *post hoc* Dunn's multiple comparisons). It should be noted that at day 0 the median IgM titers of both immunization groups and the nonvaccinated control group were at least 20-fold increased compared to the respective median IgA and IgG1 titers (Fig. 4 and 5). The median IgM titers peaked at day 14 or 28 in both immunization groups followed by slight to moderate declines, most likely due to isotype switching (Fig. 4A and 5A). In contrast, the median IgA and IgG1 antibody titers to 2019-R OMVs (Fig. 4B and C) or 3198-R OMVs (Fig. 5B and C) of both immunization groups increased during the immunization period.

**Figure 6:**
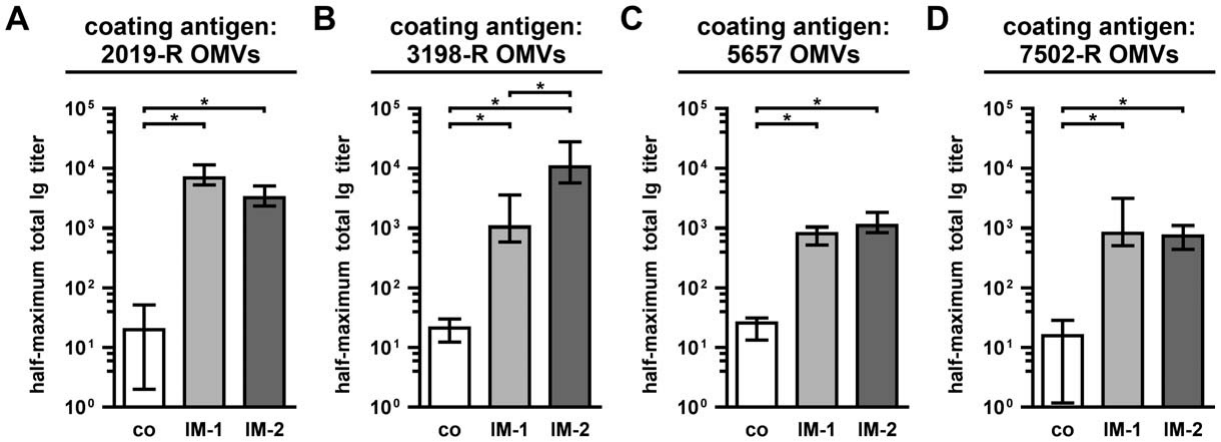
Half-maximum total Ig titers to OMVs derived from homologous and heterologous NTHi strains. Depicted are the median half-maximum total Ig titers to OMVs derived from NTHi strains 2019-R (A), 3198-R (B), 5657 (C), and 7502-R (D) in sera collected at day 39 from mice intranasally immunized with either IM-1 or IM-2 as well as from nonvaccinated control mice (co) (n = 20 for each group). The error bars indicate the interquartile range of each data set. Significant differences between the data sets are marked by asterisks (*P*<0.05; Kruskal-Wallis test and *post hoc* Dunn's multiple comparisons).

We also determined the half-maximum total Ig titers in sera collected at day 39 from mice of both immunization groups as well as from nonvaccinated control mice to homologous and heterologous OMVs derived from the NTHi strains 2019-R, 3198-R, 5657, and 7502-R (Fig. 6). The half-maximum total Ig titers allowed us to simultaneously detect IgM, as well as abundant subclasses of IgG and IgA and therefore served as important immunological reference values to determine the total immunogenicities of the immunization mixtures. In all cases, the half-maximum total Ig titers of mice immunized with either IM-1 or IM-2 were significantly higher than those of the nonvaccinated control mice (*P*<0.05; Kruskal-Wallis test and *post hoc* Dunn's multiple comparisons). Depending on the OMV type coated on the ELISA plates, the median half-maximum total Ig titers of the immunization groups were 30- to 500-fold higher for 5657 and 3198-R (Fig. 6C and B), respectively, if compared to the nonvaccinated control group.

**Figure 7:**
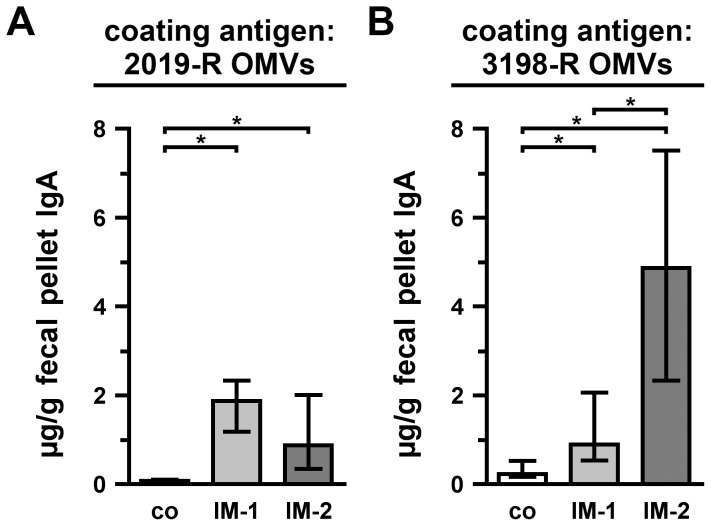
Mucosal immune responses to OMVs derived from NTHi strain 2019-R and 3198-R. Shown are the median IgA titers to OMVs derived from NTHi strain 2019-R (A) and 3198-R (B) extracted from fecal pellets collected at day 39 from mice intranasally immunized with either IM-1 or IM-2 as well as from nonvaccinated control mice (co) (n = 10 for each group). The error bars indicate the interquartile range of each data set. Significant differences between the data sets are marked by asterisks (*P*<0.05; Kruskal-Wallis test and *post hoc* Dunn's multiple comparisons).

In addition to the humoral immune responses, we also determined the induced mucosal immune responses by measuring the secretory IgA titers in fecal pellet extracts collected at day 39 from mice of both immunization groups as well as from nonvaccinated control mice to OMVs derived from either the homologous NTHi strain 2019-R (Fig. 7A) or the heterologous NTHi strain 3198-R (Fig. 7B). Both immunization groups showed significantly higher median secretory IgA titers to 2019-R and 3198-R OMVs compared to the nonvaccinated control group (Fig. 7; *P*<0.05; Kruskal-Wallis test and *post hoc* Dunn's multiple comparisons).

### OMVs contain numerous proteins that can serve as antigens

**Figure 8:**
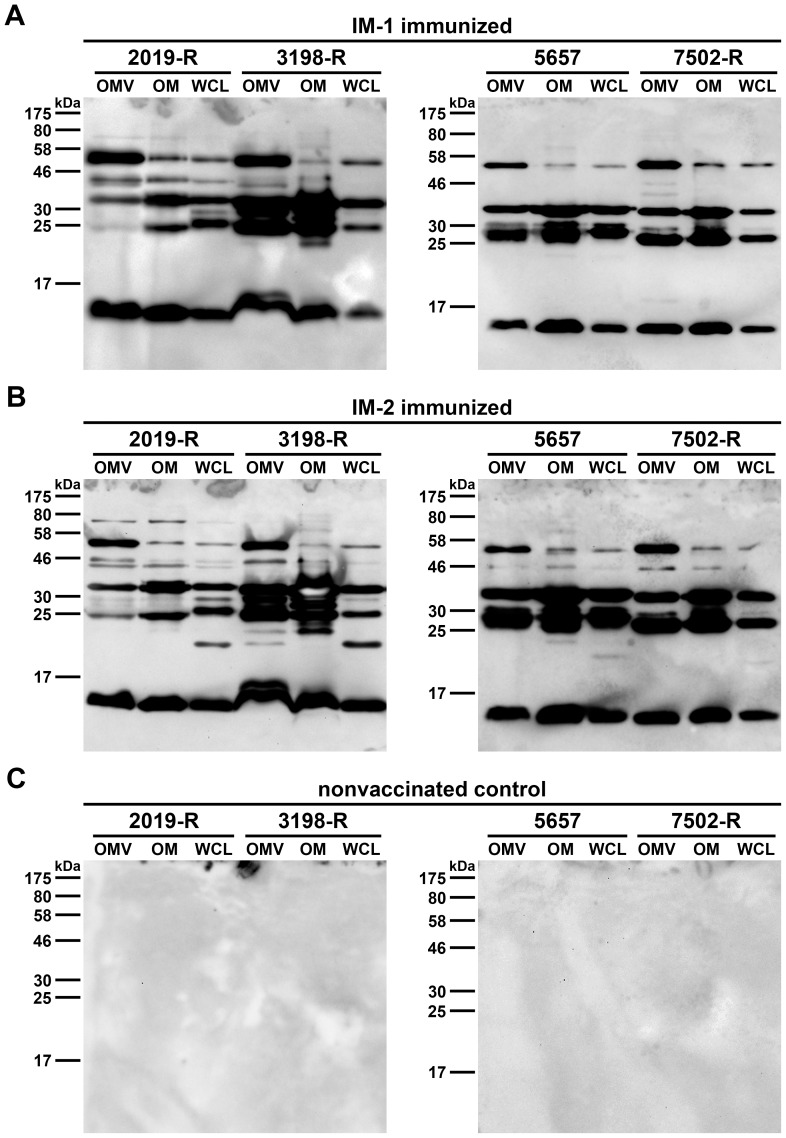
Immunoblot analysis of IgG reactivity in sera from intranasally immunized mice. Representative immunoblots incubated with sera collected at day 39 from mice intranasally immunized with either IM-1 (A) or IM-2 (B) as well as from a nonvaccinated control mouse (C) are shown. Each immunoblot was loaded with OMV, OM, and WCL preparations (approx. 3.2 µg protein each) derived from either NTHi strain 2019-R and 3198-R or 5657 and 7502-R. Lines to the left indicate the molecular masses of the protein standards in kDa.

To test the specificity of the antibody response, immunoblot analyses using OMV, OM, and WCL preparations derived from the NTHi strains 2019-R, 3198-R, 5657 and 7502-R as antigens were performed. To analyze the IgG reactivity, immunoblots were incubated with sera collected at day 39 from one mouse either immunized with IM-1 (Fig. 8A) or IM-2 (Fig. 8B) as well as from a nonvaccinated control mouse (Fig. 8C). No bands were detected on immunoblots using sera from nonvaccinated control mice (Fig. 8C). In contrast, multiple bands in the OMV, OM, and WCL protein profiles of the homologous NTHi strain 2019-R and even the heterologous NTHi strains 3198-R, 5657 and 7502-R were detected by using sera from the immunized mice (Fig. 8A and B). This clearly demonstrates that the OMVs used in IM-1 and IM-2 contain numerous proteins that can serve as antigens. In general, the most reactive bands are located at approx. 50, 35, 25, and 15 kDa.

**Figure 9:**
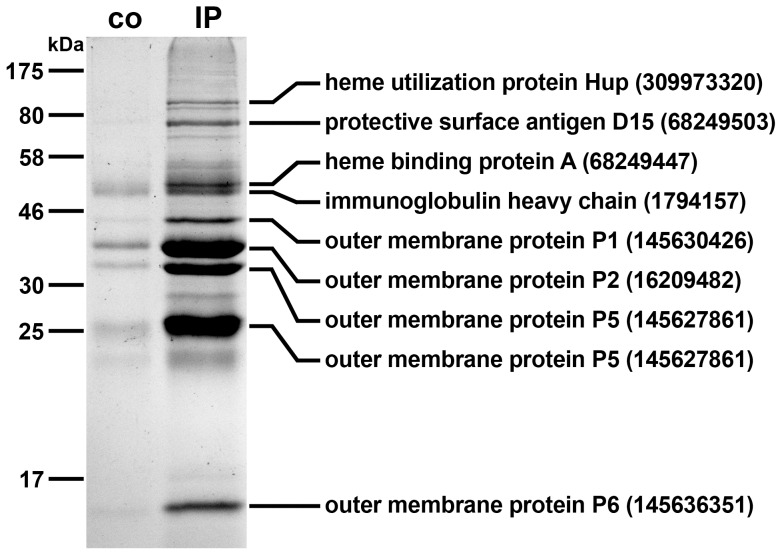
Immunoprecipitation using pooled sera from intranasally IM-1 immunized mice. Kang stained gel showing OMPs that co-immunoprecipitate with serum antibodies from nonvaccinated control mice (co) or antibodies from mice immunized with IM-1 (IP) immobilized onto Dynabeads coupled with protein G. Proteins identified from the IP sample by mass spectrometry are indicated with their respective position on the gel, protein identities and accession numbers on the right.

To identify some immunogenic proteins of NTHi OMVs, we performed immunoprecipitation analyses using sera collected at day 39 from IM-1 immunized mice as antibodies and OM preparations of strain NTHi strain 2019-R as target antigens. Sera from nonvaccinated control mice served as a negative control. The corresponding SDS-PAGE profiles of the immunoprecipitates are shown in Fig. 9. Besides the heavy chain mouse immunoglobulin migrating at approximately 50 kDa, all the other protein bands appeared to be more intensive in the immunoprecipiation using sera from immunized mice compared to the nonvaccinated control mice. The faint bands present in the control could be explained by intrinsic level of natural IgM antibodies to NTHi, which also bind to the protein G used for the immunoprecipitation. Their presence is indicated by the relatively high initial IgM titers (Fig. 4 and 5), which have not been detected in immunizations studies analyzing OMVs derived from *V. cholerae* as a vaccine candidate [34], [35]. Additionally, we cannot exclude that the OM preparations used in the immunoprecipitation contained protein complexes, which were not completely dissociated and could have been pulled down as aggregates. That is why we only focused on the most intensive protein bands of the immunoprecipiation using sera from immunized mice. These bands were excised and subjected to mass spectrometry. This analysis elucidated the seven immunogenic proteins that are indicated with their respective position in the gel provided in Fig. 9. Amongst others, the protective surface antigen D15 as well as the OMPs P2, P5 and P6 were identified to be important antigens of this NTHi vaccine candidate based on OMVs.

### Immunization with NTHi OMVs induces cross-protective immunity

**Figure 10:**
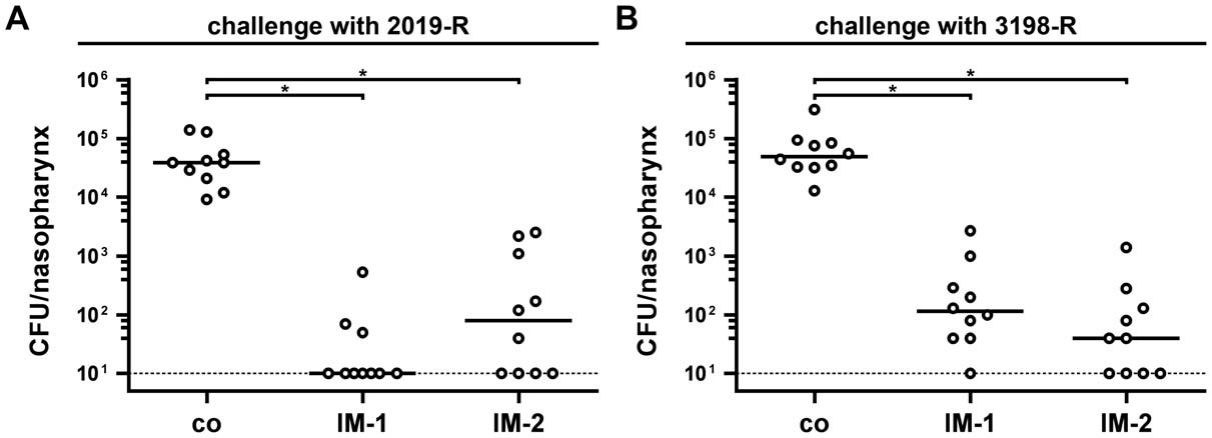
Induced immune responses in mice intranasally immunized with NTHi OMVs are protective against nasopharyngeal challenge. Shown are the nasopharyngeal colonization rates in recovered CFU per nasopharynx for mice intranasally immunized with either IM-1 or IM-2 as well as for nonvaccinated control mice (co). Mice were intranasally challenged with either NTHi strain 2019-R (A) or 3198-R (B) for 24 h. Each circle represents the recovered CFU per nasopharynx from one mouse. The horizontal bars indicate the median of each data set. If no bacteria could be recovered, then the values were set to the limit of detection of 10 CFU/nasopharynx (indicated by the dotted line). Since independent immunization rounds were performed, the exact infection doses ranged from 3.3×10^5^ to 6.0×10^5^ CFU/mouse for NTHi strain 2019-R and from 4.1×10^5^ to 4.3×10^5^ CFU/mouse for NTHi strain 3198-R. Significant differences between the data sets are marked by asterisks (*P*<0.05; Kruskal-Wallis test and *post hoc* Dunn's multiple comparisons).

In order to investigate whether the induced immune responses were protective against NTHi colonization of the mouse nasopharynx, we challenged all vaccinated mice, which were intranasally immunized with either IM-1 or IM-2, as well as all nonvaccinated control mice with the NTHi strains 2019-R or 3198-R. Therefore, all groups were divided into two subgroups and challenged intranasally with either the homologous NTHi strain 2019-R or the heterologous NTHi strain 3198-R at day 38. To determine the right infection doses of these NTHi strains, preliminary experiments were performed demonstrating that an infection dose of approximately 10^4^ CFU/mouse is just sufficient for a stable colonization of both strains over 24 h (data not shown). To ensure a stable colonization, we challenged the mice with a 50-fold higher infection dose of about 5×10^5^ CFU/mouse. Fig. 10 shows the nasopharyngeal colonization rates in recovered CFU per nasopharynx for all vaccinated and nonvaccinated control mice after challenge with NTHi strain 2019-R (Fig. 10A) or 3198-R (Fig. 10B) for 24 h. All nonvaccinated control mice were stably colonized with median colonization rates of 4×10^4^ or 5×10^4^ CFU/nasopharynx for NTHi strains 2019-R or 3198-R, respectively. In contrast, both immunization groups challenged with either NTHi strain 2019-R or 3198-R showed significant reductions in their nasopharyngeal colonization rates compared to the nonvaccinated control group (*P*<0.05; Kruskal-Wallis test and *post hoc* Dunn's multiple comparisons). For both challenge experiments, no significant differences in the colonization rates between the IM-1 and the IM-2 group were observed. The intranasal immunization with IM-1 resulted in 3,900-fold (challenge with 2019-R) and 400-fold (challenge with 3198-R) reduced median colonization rates, whereas the intranasal immunization with IM-2 led to 500-fold and 1,200-fold reductions in the median colonization rates of NTHi strain 2019-R and 3198-R, respectively. Several immunized mice showed no detectable colonization at all. In these cases, the colonization rates were set to the limit of detection of 10 CFU/nasopharynx. In summary, the induced immune responses in mice immunized with either IM-1 or IM-2 are not only protective against a homologous NTHi strain, but also against a heterologous NTHi strain, whose OMVs were not present in the immunization mixtures.

### The protective immunity correlates with intranasal immunization and the use of NTHi OMVs

**Figure 11:**
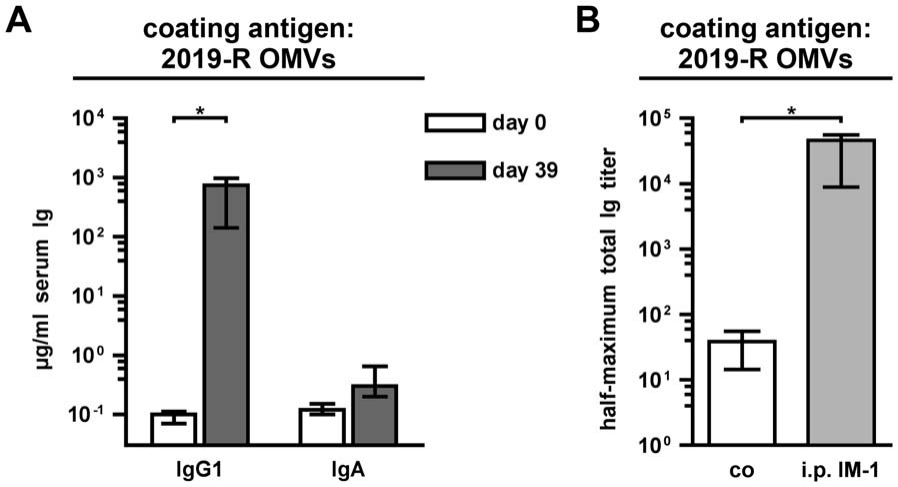
Characterization of the immune response from mice intraperitoneally immunized with NTHi OMVs. (A) Shown are the median IgG1and IgA titers to OMVs derived from NTHi strain 2019-R in sera from mice intraperitoneally immunized with IM-1 collected at day 0 and 39 (n = 7). (B) Shown are the median half-maximum total Ig titers to OMVs derived from NTHi strains 2019-R in sera collected at day 39 from mice intraperitoneally immunized with IM-1 (i.p. IM-1) as well as from nonvaccinated control mice (co) (n = 6 for the co group and n = 7 for the i.p. IM-1 group). The error bars indicate the interquartile range of each data set for each time point. Significant differences between the data sets are marked by asterisks (*P*<0.05; Mann-Whitney U test).

**Figure 12:**
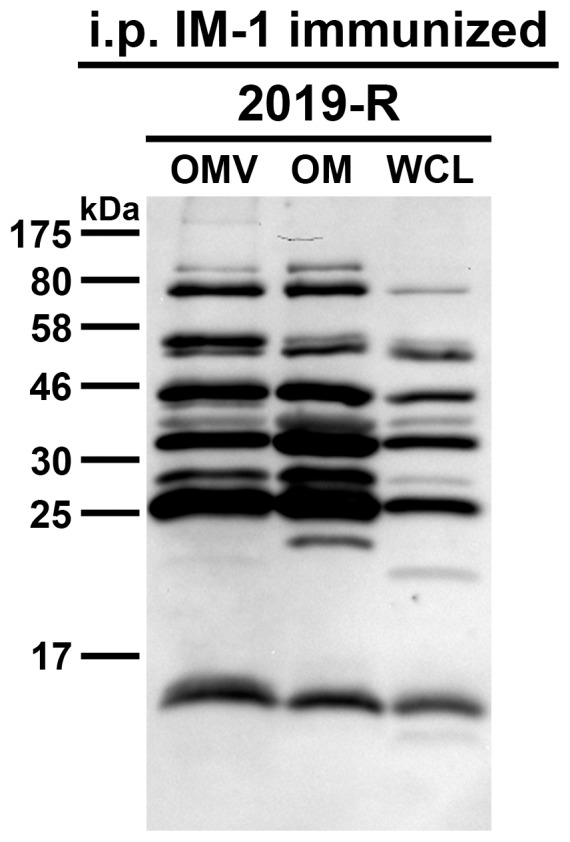
Immunoblot analysis of IgG reactivity in sera from intraperitoneally immunized mice. Shown is a representative immunoblot incubated with sera collected at day 39 from a mouse intraperitoneally immunized with IM-1 (i.p. IM-1). The immunoblot was loaded with OMV, OM, and WCL preparations (approx. 3.2 µg protein each) derived from NTHi strain 2019-R. Lines to the left indicate the molecular masses of the protein standards in kDa.

**Figure 13:**
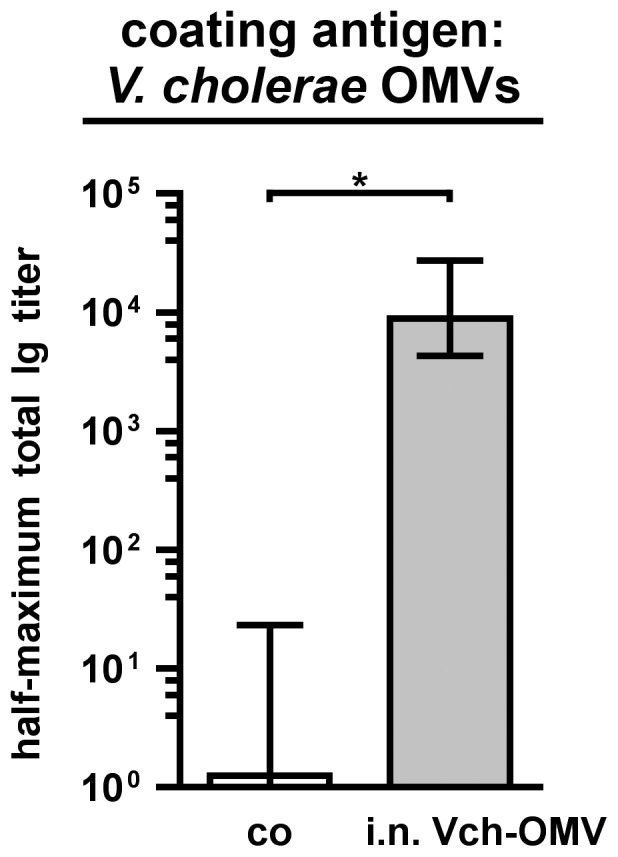
he protective immune responses correlates with intranasal immunization using species-specific OMVs. Shown are the nasopharyngeal colonization rates in recovered CFU per nasopharynx for mice intraperitoneally immunized with NTHi 2019-R (i.p. IM-1) or intranasally with OMVs derived from *V. cholerae* (i.n. Vch-OMV) as well as for nonvaccinated control mice (co). Mice were intranasally challenged with NTHi strain 2019-R for 24 h. Each circle represents the recovered CFU per nasopharynx from one mouse. The horizontal bars indicate the median of each data set. The limit of detection for this experiment was 10 CFU/nasopharynx (indicated by the dotted line). The exact infection doses in the independent experiments were approximately 2×10^5^ CFU/mouse. Significant differences between the data sets are marked by asterisks (*P*<0.05; Kruskal-Wallis test and *post hoc* Dunn's multiple comparisons).

In addition, we performed two complementary immunization experiments to characterize the protective immune response based on NTHi OMVs as vaccine candidates in more detail. Previous immunizations studies using the mouse model and human epidemiological data on otitis media caused by NTHi suggest that IgA, especially secretory IgA in the mucosal surfaces, might be the most important isotype for enhanced clearance of NTHi infections [19], [75], [76]. Mucosal immunizations like intransasal administration result in a robust IgA response, whereas immunization via the intraperitoneal route does not induce high IgA levels [34], [57], [77], [78], [79]. We took advantage of this difference and intraperitoneally immunized mice using OMVs derived from NTHi strain 2019-R (i.p. IM-1). Concordant with the current literature, the intraperitoneally immunized mice induced a robust IgG1 response to OMVs derived from NTHi strain 2019-R, but showed only a small, insignificant induction in IgA levels (Fig. 11A). We also determined the half-maximum total Ig titers in sera collected at day 39 (Fig. 11B). As expected, the half-maximum total Ig titers in sera of the intraperitoneally immunized mice were significantly higher than those of the nonvaccinated control mice (*P*<0.05; Mann-Whitney U test). Similar levels in the half-maximum total Ig titers detected in mice immunized with IM-1 via the intransal or intraperitoenal route indicate that both immunization strategies induced a robust immune response with comparable total antibody levels (Fig. 6A and 11B). In addition, the specificity of the IgG response was analyzed by immunoblot analysis (Fig. 12). Multiple bands in the OMV, OM, and WCL protein profiles derived from the NTHi strain 2019-R were detected using sera collected at day 39 from a representative mouse intraperitoneally immunized with IM-1. Thus, the immune response induced upon intraperitoneal immunization is most likely as diverse as upon intranasal immunization and many components of the OMVs serve as antigens. We challenged the intraperitoneally IM-1 immunized mice with NTHi strain 2019-R and found a significant 25-fold reduction in the median colonization rate compared to the nonvaccinated control group (Fig. 13, *P*<0.05; Kruskal-Wallis test and *post hoc* Dunn's multiple comparisons). In contrast to the group receiving IM-1 intranasally, all intraperitoneally immunized mice were colonized with detectable levels and 3-log reduction rates upon challenge with NTHi strain 2019-R could not be reached (Fig. 10 and 13). Thus, the intranasal route of immunization with NTHi OMVs confers higher protection than the intraperitoenal route.

**Figure 14:**
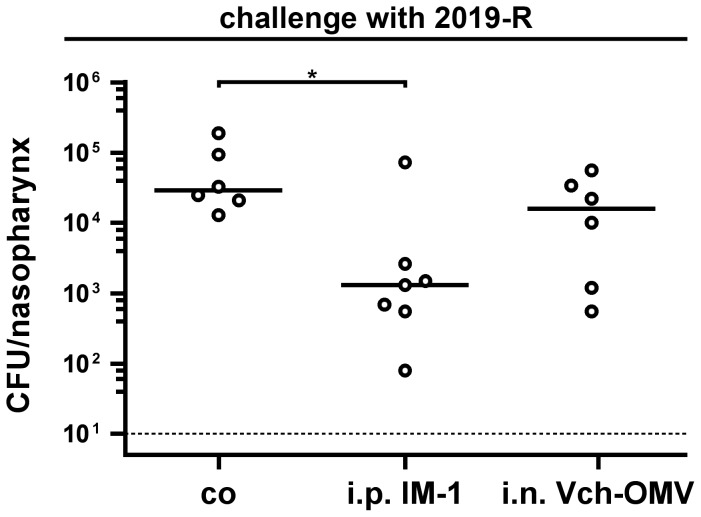
Half maximum total Ig titers in sera from mice intranasally immunized with *V. cholerae* OMVs. Depicted are the median half-maximum total Ig titers to OMVs derived from *V. cholerae* in sera collected at day 39 from mice intranasally immunized with *V. cholerae* OMVs as well as from nonvaccinated control mice (co) (n = 6 for each group). The error bars indicate the interquartile range of each data set. Significant differences between the data sets are marked by asterisks (*P*<0.05; Mann-Whitney U test).

It is very likely that the intranasal immunization with OMVs results in local inflammation and recruitment of phagocytes to the nasopharynx. Thus, such general, unspecific responses of the innate immune system could also account for the observed protection. In order to investigate the species specificity of the immune response we performed intranasal immunization studies according to the immunization schedule with OMVs derived from *V. cholerae* (i.n. Vch-OMV), which have been shown recently to be highly immunogenic and induce a protective immune response against this gastrointestinal pathogen [34], [35]. Consistent with these previous studies, the mice induced a robust immune response against *V. cholerae*, which is indicated by a 7000-fold higher median half-maximum total Ig titer detected in sera of the *V. cholerae* OMV immunized mice compared to the nonvaccinated control group (Fig. 14). In contrast, no significant immune response was induced against NTHi strain 2019-R, which was monitored by determination of the half-maximum total Ig titers and immunoblot analysis using NTHi strain 2019-R OMVs as antigen (data not shown). Challenge of the *V. cholerae* OMV immunized mice with NTHi strain 2019-R revealed only a minor, insignificant 2-fold reduced median colonization rate compared to the nonvaccinated control group (Fig. 13). Thus, the 2- to 3-log reduction of the colonization rates after intranasal immunization with NTHi OMVs could not be reached. In summary, the additional immunization experiments indicate that observed protection against nasopharyngeal colonization with NTHi upon immunization with OMVs highly correlates with the intranasal administration route as well as the use of species-specific OMVs.

## Discussion

The major aim of the present study was to investigate the potential of OMVs derived from NTHi strains to serve as a future vaccine against NTHi infections. OMVs derived from the NTHi strains used in this study were characterized by electron microscopy as well as by OMV and OM profile comparisons. Additionally, we identified eight abundant proteins packaged in the vesicles. Consistent with our analysis, these proteins have also been found in the recently published proteome of OMVs derived from the NTHi strain 86-028NP with the OMPs P2 and P5 being the most abundant proteins [44]. Therefore, this study confirms the production of OMVs by heterologous NTHi strains and provides a protein profile analysis of OMVs released from multiple NTHi strains.

To test the immunogenic and protective properties of NTHi OMVs, we intranasally immunized mice either with IM-1, composed of OMVs derived solely from NTHi strain 2019-R, or with IM-2, an OMV mixture consisting of OMVs derived from NTHi strains 2019-R, 9274-R, and 1479-R. The OMV mixture provided in IM-2 was used to increase the antigen complexity of the NTHi vaccine candidate. We primarily focused on the intranasal route, since nasal immunization is the most effective route to induce a protective immunity in both systemic and mucosal sites [80], [81]. This is especially true for the upper respiratory tract [81], which is the primary site of colonization and infection by NTHi [1], [3].

Humoral and mucosal immune responses of both immunization groups were monitored by ELISA. In these assays NTHi strain 2019-R was used to determine the immune responses against a homologous strain, whose surface antigens (OMVs) were presented to the immune systems of mice intranasally immunized with IM-1 and IM-2. In contrast, OMVs derived from the heterologous NTHi strain 3198-R were not present in IM-1 or IM-2. It has to be noted that strain 3198-R was allocated into the same LOS group as 9274-R, which was used as a donor for OMVs in IM-2. Nevertheless both strains exhibit distinct differences in the OM and OMV protein profiles. Thus, the immune systems of mice immunized with IM-2 could have had contact to a similar LOS structure, but not to the same composition of surface protein antigens. In the case of the mice immunized with IM-1, NTHi strain 3198-R served as an unknown strain, whose surface-exposed protein and LOS antigens were never seen by the immune systems. In summary, this experimental design allowed us to determine and analyze the immune response against a homologous strain, against a strain with similar LOS structure, but heterologous protein profile, and against a strain with heterologous protein profile and diverse LOS structure (see Table 1 for details).

The levels of the induced immune responses were similar to other successful OMV vaccination studies using OMVs derived from *V. cholerae* or *N. meningitidis* serogroup B [34], [35], [57]. The humoral IgA and IgG1 responses steadily increased during the whole immunization period, suggesting that the maximum antibody titers were not reached at day 39. Interestingly, the high initial IgM titers have not been detected in comparable studies using OMVs derived from *V. cholerae* [34], [35]. But the BALB/c mice, which were used in the present study, were not raised under germfree conditions and thus could have been in contact with or colonized with closely related bacterial species like other *Pasteurellaceae* family members. Since NTHi belongs to the *Pasteurellaceae* family, it could be suggested that, due to the family relationship, a certain intrinsic level of low-affinity, cross-reacting, natural IgM antibodies to NTHi already exists in BALB/c mice. This assumption is supported by the fact that naïve animals produce natural antibodies that mainly belong to the IgM subclass [82], [83].

Besides the humoral immune responses, we also determined the induced mucosal immune responses by measuring the secretory IgA titers in fecal pellet extracts. The observed induction of a mucosal immune response against homologous and heterologous NTHi strains is quite an important feature of a potential NTHi vaccine, since the nasopharyngeal mucosa is the first line of defense against respiratory pathogens, such as NTHi [80], [81]. In general, secretory antibodies to NTHi are detected in body fluids that are obtained either by performing nasal washes using PBS or by collecting saliva after injection of pilocarpine to induce salivary secretion [19], [84]. However, all mice in this study were intranasally challenged with NTHi, so that nasal washes or pilocarpine injections most likely would have interfered with nasopharyngeal colonization. Therefore, we used fecal pellets to determine the mucosal immune responses, because it has been shown before that secretory IgA antibodies reflecting the mucosal immune response can also be found in feces and that IgA titers in feces correlate with those in saliva [35], [85], [86].

By comparing the elicited humoral and mucosal immune responses as well as the half-maximum total Ig titers against the homologous NTHi strain 2019-R, it became obvious that the use of an OMV mixture in IM-2 is no disadvantage for the induction of a high-titer immune response, although just a third of the amount of 2019-R OMVs were present in this immunization mixture compared to IM-1. Furthermore, mice immunized with IM-2 exhibited higher immune responses against the heterologous NTHi strain 3198-R compared to the IM-1 immunized group throughout the entire study. IM-2 contained OMVs derived from NTHi strain 9274-R, which belongs to the same LOS group as NTHI strain 3198-R. Thus, mice immunized with IM-2 might have raised antibodies against type III LOS structures. This would consequently result in a cross-reaction with LOS of NTHI 3198-R and may explain the observed immune response patterns against NTHi strain 3198-R. Therefore, we also determined the half-maximum total Ig titers against the NTHi strains 5657 and 7502-R, which belong to the LOS groups IV and V, respectively. Although neither IM-1 nor IM-2 contained OMVs derived from a type IV or V NTHi strain, mice of both immunization groups showed a significant immune response against these strains. This indicates that intranasal immunization with OMVs derived from just one NTHi strain is sufficient to induce a robust humoral immune response against heterologous and unknown NTHi strains.

Immunoblot analyses were performed to characterize the complexity of the induced immune response. Multiple bands in the OMV, OM, and WCL protein profiles were detected not only in preparations derived from the homologous NTHi strain 2019-R, but also in preparations derived from the heterologous NTHi strains 3198-R, 5657, and 7502-R. Similar results have also been reported by immunization studies using OMVs derived from *V. cholerae* and *Acinetobacter baumannii* [34], [87]. Interestingly, already mice immunized with IM-1, just containing one OMV type, induced a quite diverse immune response against variety of antigens including even those from heterologous NTHi strains unknown to the immune system. Immunoprecipitation revealed several important immunogenic proteins. Heme utilization protein and heme binding protein A are both involved in heme utilization, which is a growth requirement for *H. influenzae* and affects virulence [88], [89], [90]. Furthermore, protective surface antigen D15 as well as the OMPs P1, P2, P5 and P6 have already been independently suggested as promising antigens for NTHi vaccine candidates [13], [14], [16], [91], [92], [93]. Thus, NTHi OMVs contain numerous immunogenic proteins and allow the combined presentation of these antigens to the host strengthening their potential as a candidate for NTHi vaccine development.

The protective capacity of the induced immune responses is highlighted by the intranasal challenge of the mice with either the homologous NTHi strain 2019-R or the heterologous NTHi strain 3198-R. Regardless of the NTHi strain challenged with, groups intranasally immunized with NTHi OMVs showed significant reductions in their nasopharyngeal colonization rates compared to the nonvaccinated control group, ranging from 400 to 3,900-fold. To our knowledge, this is the first mouse study reporting such high reduction rates for nasopharyngeal colonization studies with NTHi after immunization with a potential NTHi vaccine. We analyzed the protective immune response in more detail and performed two control immunization experiments. We changed the administration route and intraperitoneally immunized mice with IM-1 to avoid induction of a IgA response. These intraperitoneally immunized group exhibited a significant reduction of the nasopharyngeal colonization, but the median colonization rate in intraperitoneally immunized mice was still 100-fold higher compared to the group intranasally immunized with NTHi OMVs. Thus, the best protection upon immunization with NTHi OMVs was observed via the mucosal administration route. Although we cannot exclude other mechanisms, it would be concordant with epidemiological studies on NTHi infections that the IgA response, especially the levels of secretory IgA, are the most important antibodies for the protection against NTHi infections [75], [76]. Besides the induction of a robust antibody response, intranasal immunizations with OMVs will most likely also stimulate antibody-independent mechanism of the immune system, i.e. inflammation, cytokine secretion and recruitment of phagocytes. To investigate the potential role of such general, unspecific responses of the innate immune system we performed intranasal immunizations using OMVs from *V. cholerae*, which is only distantly related to NTHi. Consequently, these mice induced a robust antibody response against *V. cholerae*, but not against NTHi. Since *V. cholerae* OMV immunized mice failed to show protection against challenge with NTHi, nonspecific mechanisms of the immune system cannot account for the reduced colonization observed upon immunization with NTHi OMVs. Consequently, the protective immune response mainly depends on the use of NTHi OMVs and is therefore specific to the OMVs present in the vaccine candidate. Recently, an antibody-independent, CD4^+^ dependent protective immunity against pneumococcal colonization was described after mucosal immunization using a pneumococcal whole cell killed vaccine [94], [95]. At the current stage similar effects, which are antibody-independent, but specific to NTHi OMVs, cannot be excluded for our model and future investigations using for example antibody-deficient mice will be necessary to investigate all facets of the protective immunity in more detail.

As mentioned above, IM-2 contained OMVs from NTHI strains allocated into the LOS groups I, II and III. Consequently, these mice could have raised immunoglobulins directed against the LOS structures of the NTHI strains 2019-R (type II) and 3198-R (type III) used for the challenge. Thus, we cannot exclude that the observed protection for the IM-2 immunization group relies on anti-LOS antibodies. However, this does not explain the robust induced immune response against heterologous NTHi strains and protection against NTHI strain 3198-R of mice immunized with IM-1, containing solely OMVs derived from NTHI strain 2019-R. Additionally, a recent study by Hirano et al. investigated the protective immune response upon immunization with a LOS-based conjugate vaccine candidate in the mouse model [19]. Immunized mice showed only a 2- to 4-fold reduction in nasopharyngeal colonization after challenge with homologous or heterologous NTHi strains compared to the control group. The higher reduction rates observed in the present study suggest that the protective immune response of the OMV-based vaccine candidate does not depend on antibodies against the LOS structures. Hence, immunization with OMVs results in cross-protection between different LOS groups and the LOS is most likely not the dominant protective antigen.

This is in contrast to a recent OMV immunization study, demonstrating that LPS is the major protective antigen of a *V. cholerae* vaccine candidate based on OMVs and that no cross-serogroup protection can be achieved by just using OMVs derived from one *V. cholerae* serogroup in the immunization mixture [32]. One explanation might be that the LOS of NTHi strains is much shorter compared to the *V. cholerae* LPS with relatively long and variable O-antigens, which might act as spacers between the surface antigens associated with the OM and the respective antibodies.

Like any other animal model, the NTHi mouse model cannot reflect all parts of the human infection. A limitation is the lack of a chronic pulmonary colonization due to rapid clearing of the bacteria in the respiratory tract. Nevertheless, the mouse model is well established in the field and has been used in the past to study several NTHi vaccine candidates [15], [19], [96]. This study should be seen as a first report characterizing a new vaccine candidate based on OMVs, which induces cross-protection against heterologous NTHi strains. Future work has to investigate, if the initial results of the present study using the mouse model also hold true in the human system. Noteworthy, in the case of otitis media caused by NTHi, several studies suggest a correlation between the presence of serum antibodies against NTHi and protection [75], [76], [97], [98].

In summary, this study has confirmed that NTHi strains release OMVs and has shown that these OMVs have a high potential to act as vaccine against NTHi infections. We have demonstrated that already an intranasal immunization with OMVs derived from one NTHi strain results in a robust and complex humoral, mucosal, and protective immune response against homologous and heterologous NTHi strains, even without the use of a mucosal adjuvant. Obviously, OMVs naturally contain a balanced mixture of immunogenic, protective antigens and adjuvants. OMVs can be isolated from different donor strains and easily combined in OMV mixtures. Based on the data of this study, the use of an OMV mixture is not necessary, but also has no disadvantage for the induction of a protective immune response against NTHi strains. It can be speculated that the use of a heterogeneous OMV mixture might be advantageous by inducing a more complex immune response against different antigens of NTHi strains. Additionally, one could extend this idea and create a combined vaccine candidate against several Gram-negative pathogens by combining OMVs derived from different donor species. Thus, the increased antigenic diversity of an OMV mixture could further stimulate the induction of cross-reacting antibodies, which in turn may help to develop a broad-spectrum vaccine not only against heterologous NTHi strains, but also against other Gram-negative pathogens of interest.
