# Correction: Intranasal Immunization with Nontypeable *Haemophilus influenzae* Outer Membrane Vesicles Induces Cross-Protective Immunity in Mice

**DOI:** 10.1371/annotation/0dbc4010-f114-42fc-aafa-3efeef4d3068

**Published:** 2012-08-14

**Authors:** Sandro Roier, Deborah R. Leitner, Jeremy Iwashkiw, Kristina Schild-Prüfert, Mario F. Feldman, Georg Krohne, Joachim Reidl, Stefan Schild

The images for Figures 1 and 2, as well as Figures 13 and 14, were incorrectly switched. The image that appears as Figure 1 should be Figure 2, and the image that appears as Figure 2 should be Figure 1. The image that appears as Figure 13 should be Figure 14, and the image that appears as Figure 14 should be Figure 13. The figure legends appear in the correct order. 

